# A Novel Carbon Nanofibers Grown on Glass Microballoons Immunosensor: A Tool for Early Diagnosis of Malaria

**DOI:** 10.3390/s140814686

**Published:** 2014-08-12

**Authors:** Emmanuel Gikunoo, Adeyabeba Abera, Eyassu Woldesenbet

**Affiliations:** 1 Mechanical Engineering Department, National Science Foundation Center for Next Generation Composites, Louisiana State University, Baton Rouge, LA 70802, USA; E-Mail: woldesen@me.lsu.edu; 2 Mechanical Engineering Department, National Science Foundation Center for Next Generation Composites, Southern University and A & M College, Baton Rouge, LA 70813, USA; E-Mail: adeyabera@gmail.com

**Keywords:** *Plasmodium falciparum* histidine rich protein-2, immunosensor, rapid diagnostic tests, ultrasensitive, malaria, carbon nanofiber

## Abstract

This paper presents a novel method for direct detection of *Plasmodium falciparum* histidine rich protein-2 (*Pf*HRP-2) antigen using carbon nanofiber (CNF) forests grown on glass microballoons (NMBs). Secondary antibodies specific to *Pf*HRP-2 densely attached to the CNFs exhibit extraordinary ability for the detection of minute concentrations of *Plasmodium* species. A sandwich immunoassay protocol was employed, where a glass substrate was used to immobilize primary antibodies at designated capture zones. High signal amplification was obtained in both colorimetric and electrical measurements due to the CNFs through specific binding. As a result, it was possible to detect *Pf*HRP-2 levels as low as 0.025 ng/mL concentration in phosphate buffered saline (PBS) using a visual signal within only 1 min of test duration. Lower limits of 0.01 ng/mL was obtained by measuring the electrical resistivity of the capture zone. This method is also highly selective and specific in identifying *Pf*HRP-2 and other *Plasmodium species* from the same solution. In addition, the stability of the labeling mechanism eliminates the false signals generated by the use of dyes in current malaria rapid diagnostic test kits (MRDTs). Thus, the rapid, sensitive and high signal amplification capabilities of NMBs is a promising tool for early diagnosis of malaria and other infectious diseases.

## 1. Introduction

Malaria is one of the most fatal infectious tropical diseases known to humans. Malaria has always been a medical emergency, especially, among children under 5 years old and pregnant women. The disease spreads mostly in places with limited resources such as in Sub-Saharan Africa, South America, and Asia [1–5]. These areas usually are also characterized by high temperature and humidity, coupled with environmental degradation. Delays in diagnosis and lack of effective treatment are leading causes of death in many malaria rife countries [6]. In addition, the development of resistance to treatments from repeated use of non-prescribed malaria drugs continues to pose challenges in management of the disease [7–11].

Approximately 300 to 500 million global cases of clinical malaria are reported yearly, with an estimated 627,000 deaths occurring in 2012 [12]. Of these deaths, children in Africa have been the most affected due to their vulnerability to infection as a result of malnutrition. Most of these deaths related to malaria can be prevented with prompt diagnosis and treatment [6]. The long-lasting experience of microscopy-based diagnostics in poor regions and settings turns it undoubtedly into a relatively simple technique, in the sense that, although still requiring trained manpower, the technique is widely known and available. The result of this is that microscopy may be more feasible and cost-effective where it has already been deployed. Moreover, microscopy is usually advantageous over malaria rapid diagnostic test kits (MRDTs) for identifying the actual infecting species (as different *Plasmodium* species imply different therapeutic options) and in the case of mixed infections. It is also especially important when a MRDT yields a negative result in a suspicious case and/or in endemic regions. So far, MRDTs have been the obvious preferred choice where microscopy is not available.

The microscopy-based approach is able to detect as few as 5 parasites/μL of blood while MRDTs are only able to detect parasitemia above 100 parasites/μL [13]. In addition to low sensitivity, MRDTs lack specificity as a result of their inability to differentiate between individual *Plasmodium* species [14–18]. *Plasmodium falciparum* and *Plasmodium vivax* are the two most fatal malaria parasites. Histidine-rich protein II (HRP-2), genus-specific of *Plasmodium* species, is localized in several cell compartments including the cytoplasm of *Plasmodium falciparum* [19]. *Plasmodium falciparum* histidine rich protein-2 (*Pf*HRP-2) can also be detected in *in vitro* culture supernatants of synchronized parasites as early as 2 to 8 h after trophozoite development [20]. The trophozoites are the ring-like morphology of *Plasmodium* species observed in stained blood films. *Pf*HRP-2 is recovered from culture supernatants as a secreted water soluble protein with large amount of the functional amino acid groups needed for subsequent biological interaction [21,22].

The key advantage of using MRDTs is the simplicity of signal detection and ease of result interpretation. They are also rapid and cost-effective. The increase use of MRDTs by a larger populace has led to the minimization of treatment based on symptoms [14,17]. However, MRDTs are mainly based on visual detection principles by incorporating dyes and enzymes which degrade over time leading to false readings [23]. MRDTs also often suffer from low signal intensity and poor quantitative discrimination of the color-formation reaction based on label accumulation [24]. Hence, the labeling mechanism is key for improved performance of MRDTs. Substitution of these dyes and enzymes with a material such as carbon nanofiber (CNF) forest grown on glass microballoons (NMBs) could provide enhanced signal identification.

The NMBs are a combination of microballoons with millions of CNFs grown on their surface. The microballoons act as substrates and light weight transporters with the CNFs providing the large surface area and material properties needed for effective biomolecule immobilization. Overall, NMBs presents a new platform for the construction of immunoassays with many detection mechanisms. They can be used in electrochemical, optical, mass sensitive, colorimetric, and immunodipstick immunoassays for inherent signal amplification. The use of NMBs in immunoassays also provides the ability to multiplex and reduce background signal from biological samples and assay components, hence reducing background noise. NMBs can also act as modifiers of the electrotransducer surfaces, creating nanostructures with improved electrochemical response. The improvement in response may come either from the enhancement of the electron transfer or the increased efficiency of biomolecule immobilization [25].

This paper establishes the successful implementation of NMBs in rapid diagnostic test kits (RDTs) for diagnosis of malaria. The NMBs conjugated with rabbit polyclonal to *Pf*HRP-2 antibody (Rb-PAbfs) were prepared and used to detect *Pf*HRP-2 with standard solutions ranging from 0.01 to 10 ng/mL. The following sections explain the details of the preparation of this immunosensor as well as the investigation of its performance and detection limit. Sensitivity and specificity of the method was evaluated by detecting *Pf*HRP-2 from a solution of *Pf*HRP-2 and *Plasmodium vivax* merozoites surface protein-1 (*Pv*MSP-1) antigen. This NMBs immunoassay approach offers healthcare providers a simple, rapid, ultrasensitive and selective tool for the early management of malaria infections.

## 2. Materials and Methods

### 2.1. Reagents and Materials

Plain S22 glass microballoons were purchased from 3M Corporation (St. Paul, MI, USA). The following chemicals were used for the study: polyethylene glycol solution (PEG, *M*_W_ = 8000), glycerol, and bovine serum albumin (BSA) were purchased from Sigma-Aldrich (St. Louis, MO, USA); phosphate buffered saline 1× solution (PBS), 1-ethyl-3-(3-dimethylaminopropyl) carbodiimide hydrochloride (EDC), and *N*-hydroxysulfosuccinimide (sulfo-NHS) obtained from Thermo Fisher Scientific (Waltham, MA, USA); *Plasmodium falciparum* histidine rich protein-2 (*Pf*HRP-2) antigen, *Pv*MSP-1, mouse monoclonal to *Pv*MSP1 antibody (Ms-MAbv), rabbit polyclonal to *Pv*MSP1 antibody (Rb-PAbv), mouse monoclonal to *Pf*HRP-2 antibody (Ms-MAbf), and Rb-PAbf were obtained from MyBioSource Inc. (San Diego, CA, USA). All biological samples were purchased in a PBS solution, pH 7.4, containing 0.1% sodium azide.

Micro glass slides were obtained from Corning Inc. (Corning, NY, USA). Tween-20 purchased from Amresco (Solon, OH, USA), NaOH, H_2_SO_4_, and H_2_O_2_ from Macron Chemicals (Center Valley, PA, USA) were used for the cleaning process. Anhydrous toluene solution from Macron Chemicals, glutaraldehyde obtained from Sigma-Aldrich (St. Louis, MO, USA), and 3-aminopropyl- triethoxysilane (APTES) from Alfa Aesar Chemicals (Ward Hill, MA, USA) were used in the amino functionalization of the glass slides.

### 2.2. Fabrication of NMBs

The surface of S22 glass microballoons were functionalized, activated and coated with Ni as described by Gao *et al.* [26]. CNFs were subsequently grown on the Ni-coated glass microballoons using a chemical vapor deposition (CVD) technique described in reference [27]. Surface functionalization of the CNFs on the NMBs was achieved by oxidizing the CNFs in air at 400 °C in a CVD furnace for an hour. The functionalization generates the carboxyl functional group onto the surface of the CNFs.

The method used to organize the CNFs into well-defined spatial orientations on microsized spheres results in the growth of several millions of the CNFs on each microballoon [27]. The microsized structure of the NMBs greatly enhances the aggregation reaction necessary for obtaining visual signals. This aggregation of the NMBs at the capture zone drastically eliminates the wrong diagnoses currently encountered in RDTs. The high reactivity of NMBs with *Plasmodium* species will also allow low parasitemia to be detected.

### 2.3. NMB-Plasmodium Species Conjugation

CNFs have very high chemical affinity for biospecies [28]. A two-step reaction approach [29] was used in preparing the surface of CNFs for covalent conjugation with Rb-PAbf and Rb-PAbv. This reaction forms an ester intermediate on the NMBs that aids the amidation reaction. The amidation reaction involves the reaction of the intermediate with the primary amine group on the surfaces of Rb-PAbf. Same procedure was followed for the covalent conjugation of NMBs with Rb-PAbv. Very strong amide bonds were formed in each of the processes [29]. PEG was then applied forming a polyethylene oxide coating on NMBs. This coating prevents non-specific binding (NSB) of unwanted proteins to the NMBs.

### 2.4. Preparation of the Glass Substrate

Glass slides were cut into rectangular shapes (1 cm × 2.5 cm) and single capture zones marked within the mid portion of the slides. The slides were first cleaned using an ultrasonic bath in deionized water containing 2% Tween-20 by volume for an hour. The washed slides were then thoroughly flushed in deionized water and immersed in an ultrasonic bath again for an hour.

Surface modification of glass with APTES reported by Tang *et al.* [30] and Tsutsumi *et al.* [31] was modified to suit our process. Slides were initially soaked in 1 M NaOH for 30 min and then rinsed with deionized water. Washed slides were soaked in piranha solution, 30% H_2_O_2_ and 70% H_2_SO_4_, at 80 °C for 2 h. They were thoroughly washed with deionized water, followed by ethanol and acetone and dried for an hour at 110 °C. The role of piranha solution was to roughen the surface of the slides for subsequent immobilization procedures. Modification by immersing slides in 5% APTES in anhydrous toluene solution for 10 min ensued immediately resulting in the formation of amino groups on the surface of the glass slides. The slides were then thoroughly rinsed with toluene and acetone before drying at 110 °C for an hour. This process removed the excess APTES and toluene solution.

Activation of the amino-functionalized slide surfaces was achieved by immersing the slides in a 3% glutaraldehyde in PBS solution for an hour. The slides were thoroughly rinsed with ethanol and deionized water to completely remove unreacted glutaraldehyde. Nitrogen gas was used to dry the slides. A solution made up of 50 μL of 1 mg/mL Ms-MAbf and 20 μL of glycerol was prepared and spotted on the capture zone of the slide. The slide was left to incubate for an hour after which it was thoroughly washed with PBS to remove Ms-MAbf. The slide was finally incubated in a 2% BSA and 0.05% Tween-20 in PBS solution for an hour in order to block all sites from NSB. A plain glass slide was also incubated in the 2% BSA and 0.05% Tween-20 in PBS solution for an hour. This was used as a control for NSB.

### 2.5. NMB-Polyclonal Antibody Conjugation

Single and double capture zones were marked on several other slides. The slides were thoroughly cleaned, modified, and activated with amino functional groups as described earlier. All capture zones were treated to prevent non-specific binding. Firstly, Ms-MAbf were immobilized on several glass slides with single capture zone marks. The capture zone was used for sandwich immunoassay investigation of *Pf*HRP-2 with concentrations in the linear range of 0.01 ng/mL and 10 ng/mL. The visual and electrochemical detection limits of the *Pf*HRP-2 were determined within this range.

Secondly, for the glass slides with double capture zones, Ms-MAbf were immobilized on the first capture zone and Ms-MAbv on the second capture zone. Same immunoassay procedure was then executed. For performance evaluation, *Pf*HRP-2 was caught at the first capture zone. Experiment was repeated now using *Pv*MSP-1 sample in PBS with concentrations ranging from 0.01 to 10 ng/mL. *Pv*MSP-1 was caught at the second capture zone.

Thirdly, solutions made up of equal amounts of *Pf*HRP-2 and *Pv*MSP-1 samples were also prepared. Concentrations in the range of 0.01 ng/mL and 10 ng/mL for both antigens in this assay confirmed that *Pf*HRP-2 was caught at the first capture zone and *Pv*MSP-1 at the second capture zone. This allows the identification of both antigens from the same mixed sample in a single assay. Scanning electron microscopy (SEM), optical microscopy, and electrical resistivity characterization were performed.

### 2.6. Characterization

A Pro-4 four point system (Lucas Labs, Gilroy, CA, USA) was used in resistivity measurements. The system was made up of a Keithley 2400, a Pro4, and a notebook with the Pro4 V1.2.4 software installed. The system uses the dual configuration test method of ASTM standard F84-99 to compensate for errors in probe spacing and errors caused by proximity to the edge of the conducting layer. A V/I measurement was taken and recorded and the subsequent resistivity was computed.

Scanning electron microscope (SEM) micrographs were obtained using a Quanta 3D FEG Dual Beam FIB/SEM and its xT Microscope Control Software (FEI, Hillsboro, OR, USA). Fluorescence studies were performed on samples using a DM RXA2 fluorescence microscope (Leica, Wetzlar, Germany) fitted with a 100× objective lens with a 1.4 N.A. Slidebook version 4.1 was used for image acquisition from a SensiCam QE camera (Cooke, Auburn Hills, MI, USA) attached to the microscope.

Optical images were obtained from a 40 CFL inverted microscope (Axiovert, Thornwood, NY, USA) fitted with a 20× objective with a 1.0 N.A. AxioVision 4.6.1.0 was used for image acquisition from an AxioCam MRc camera attached to the microscope. Percentage area covered by the NMBs on the glass slides were determined using the ImageJ 1.46r software (Wayne Rasband, National Institutes of Health, USA).

## 3. Results and Discussion

### 3.1. Principle of Disease Diagnosis

Most MRDTs currently employ the immunochromatographic strip assay technique. This technique allows for separation and identification of soluble antigens in solution using complementary antibodies. The technique allows for mass production of kits, which in turn allows lower manufacturing and distribution cost, and a simplified supply chain. The strip assay technique also allows for easy training of users, standardization of result interpretation, and ease of use in countries or regions where multiple languages are spoken [32].

Similarly, the NMBs detection technique is based on the immunochromatography sandwich approach. As a result of specific binding between the capture antibody and antigen, visible signal is generated from the NMBs conjugated with secondary antibody. The optical signal is assessed using the naked eye and quantified using a reading device. Immobilized NMBs on the capture zone can also be quantified using the change in electrical resistivity of that capture zone.

**Figure 1.**
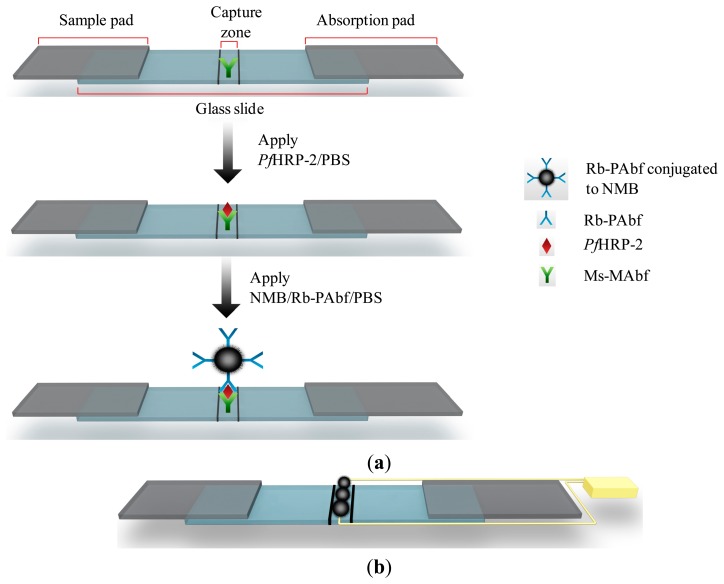
(**a**) Schematic illustration of the sandwich immunochromatographic set-up used to detect *Pf*HRP-2 and (**b**) Illustration of the two different types of signal generation from the immunosensor. The yellow wire is used to show the connection for electrical measurement.

The success of the sandwich immunoassay test requires the use of an antigen large enough to contain at least two epitopes [33]. *Pf*HRP-2, which satisfies this requirement, has two epitopes specific to Ms-MAbf and Rb-PAbf. The primary monoclonal antibody, Ms-MAbf, was immobilized on the capture zone of the glass slide as shown in Figure 1a. *Pf*HRP-2 in PBS was applied to the sample pad of the strip. Extra PBS was introduced and acts as a lysing agent to improve the sample flow to the absorption pad. The sample mixture migrated across the membrane to the capture zone, where Ms-MAbf immobilized on the strip surface captured most of the *Pf*HRP-2 present in the mixture. The absorption pad regulated the flow and collected all excess fluid. A second liquid sample of secondary polyclonal antibodies, Rb-PAbf, coupled to NMBs in PBS is applied to the sample pad. The Rb-PAbf and NMBs conjugate bind to different epitopes on the captured *Pf*HRP-2 antigens. As a result, the Rb-PAbf and NMBs conjugate captured by the *Pf*HRP-2 generated a dark line at the capture zone, giving a positive test result as shown in Figure 1b. Electrical resistivity of the capture zone can also be obtained as shown in Figure 1b.

### 3.2. Synthesis of NMBs

**Figure 2.**
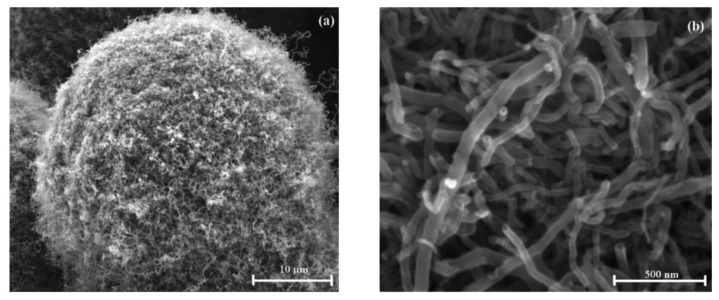
SEM micrographs of air oxidized NMBs at different magnifications.

SEM micrographs of the air-oxidized NMBs are shown in Figure 2. Several millions of the CNFs were uniformly grown on microballoons [27]. At higher magnifications, the CNFs were seen to have an average diameter of 50 nm and they were several micrometers in length. The oxidation process generated the carboxyl functional groups onto the surface of the CNFs. The CNFs forest with high aspect ratio and high chemical affinity offered several binding sites on which Rb-PAbf were immobilized. Due to the several millions of CNFs grown on each microballoon, the probability of conjugating the Rb-PAbf on the NMBs is high. Large amounts of both amino and amine functional groups on the surface of Rb-PAbf permitted their covalent bonding with the carboxylated CNFs on the NMBs. This provided NMBs with ultra-low limit of detection for *Pf*HRP-2.

### 3.3. Detection Limit of PfHRP-2 Assay

**Figure 3.**
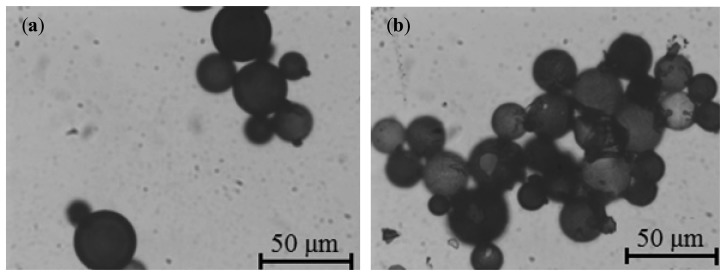
Optical micrographs depicting NMBs captured on glass slides in a sandwich assay format with (**a**) 0.025 ng/mL and (**b**) 10 ng/mL of *Pf*HRP-2.

Successful immobilization of NMBs on the capture zone of the glass slide was achieved within one minute. NMBs were observed to form an interconnecting network at the capture zone as shown in Figure 3. This confirmed the detection of *Pf*HRP-2 by the sandwich immunochromatography method. Increasing *Pf*HRP-2 concentration increased the density of the immobilized NMB network at the capture zone. This is depicted in Figure 3a for the lowest visible *Pf*HRP-2 concentration of 0.025 ng/mL and Figure 3b for the highest *Pf*HRP-2 concentration studied.

**Figure 4.**
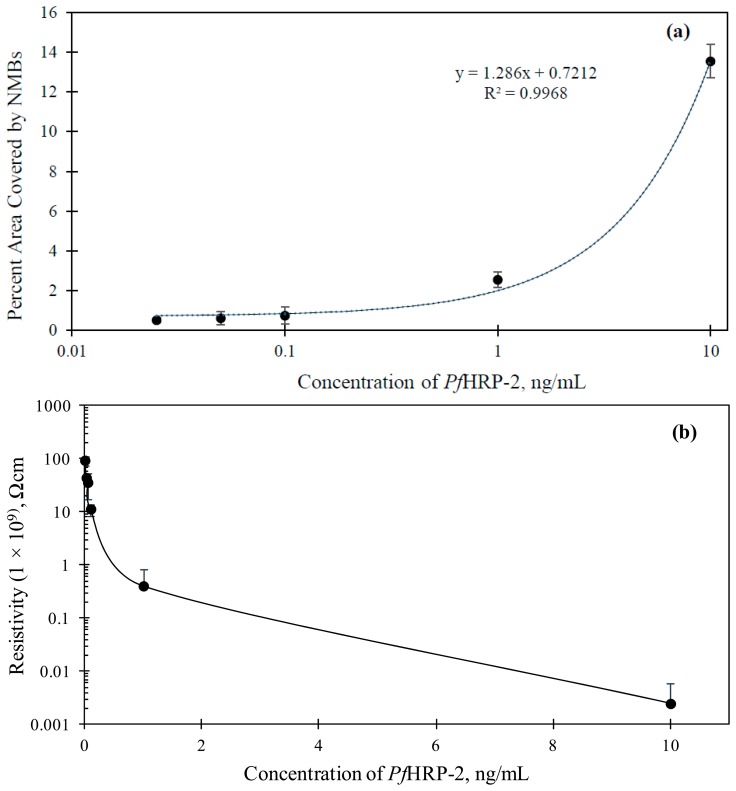
(**a**) Variation of percent area of the capture zone covered with NMBs against *Pf*HRP-2 concentrations and (**b**) Variation of electrical resistivity of the capture zone with immobilized *Pf*HRP-2 concentration. The error bars in both graphs are also shown.

A linear relationship between the percentage area covered by the NMBs (*A*_pct_) at the capture zone and the concentration of *Pf*HRP-2 in the range of 0.01 to 10 ng/mL was attained (shown in Figure 4a). The correlation equation, *A*_pct_ = 0.7212 +1.286 *x* (*R*^2^ = 0.9968) was used, where *x* is the concentration of *Pf*HRP-2. This equation can be used to estimate the concentration of *Plasmodium falciparum* in any sample tested. The visual detection limit of the assay was 0.025 ng/mL. This is the lowest concentration at which the NMB network was observed with the unaided human eye.

The lowest detection limit of *Pf*HRP-2 reported in the literature to date using immunoassay technique has been 0.36 ng/mL [34]. The ultra-low visual detection limit of 0.025 ng/mL obtained from this study is attributed to the following. First, the high aspect ratio of CNFs makes available several binding sites for covalent bonding with Rb-PAbf. The high aspect ratio arises from the construction of the CNFs giving them length-to-diameter ratio of up to 132,000,000:1. This high ratio gives CNFs extensive area on their length for several of the nanosized antigens to be immobilized. Second, the exploitation of the high chemical reactivity of CNFs with antibodies of *Pf*HRP-2 and organic surfactants make it possible for CNFs to be highly selective and specific in aqueous environment. Third, large effective surface area remains available on each micro-structured NMB allowing it to be treated with several millions of *Pf*HRP-2 antibodies. Finally, the microstructure of the NMBs allows visual signal amplification and hence easier quantification.

Signal detection and quantification of this immunosensor can also be achieved using electrical measurement. The high conducting nature of CNFs [27] provides the flexibility to perform resistivity changes on the capture zone of the immunosensor in addition to the visual characterization explained earlier. The interconnected pathway formed by the immobilized NMBs at the capture zone allowed for an effective electron transfer to be established with the electrode. The resistivity of the capture zone was measured at different *Pf*HRP-2 concentrations as shown in Figure 4b. The higher the concentration of *Pf*HRP-2, the higher was the density of the immobilized NMBs and hence the more the CNFs available for conduction. Resistivity of the plain glass slide used for the study was 9.30 × 10^10^ Ω·cm. Resistivity measurement was also conducted in NSB case and it was measured as 8.99 × 10^10^ Ω·cm. Appreciable change/reduction in resistivity was only observed for concentrations of *Pf*HRP-2 0.01 ng/mL and greater. This set a limit of detection of 0.01 ng/mL, which is considered very low.

### 3.4. Selectivity of the PfHRP-2 Detection Assay

**Figure 5.**
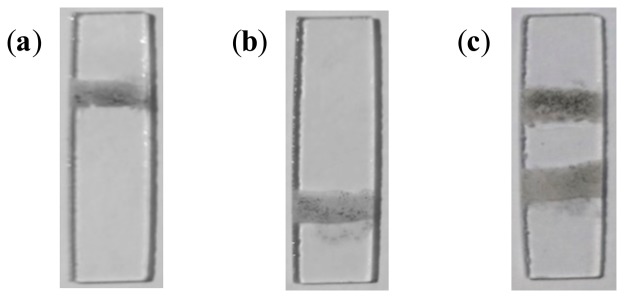
Glass slides showing (**a**) visual signal obtained at the first capture zone from the detection of *Pf*HRP-2 in PBS; (**b**) signal observed at the second capture zone from the detection of *Pv*MSP-1 in solution; and (**c**) signals obtained at the *Pf*HRP-2 and *Pv*MSP-1 capture zones from a mixed *Pf*HRP-2 and *Pv*MSP-1 solution.

The selectivity of the proposed method was also established. In order to determine this capability, two capture zones were spotted on the immunosensor. On the first capture zone, primary antibodies specific to *Pf*HRP-2 were immobilized. Antibodies specific to *Pf*MSP-1 were immobilized on the second capture zone, using the method described earlier. Figure 5a shows the immunosensor for the detection of 10 ng/mL of *Pf*HRP-2 in PBS solution. Quantification of the percent area covered by captured NMBs showed that increasing antigen concentration increases the amount of NMBs immobilized at the capture zone. During the tests with various concentrations of *Pf*HRP-2, no signal was observed at the second capture zone.

**Figure 6.**
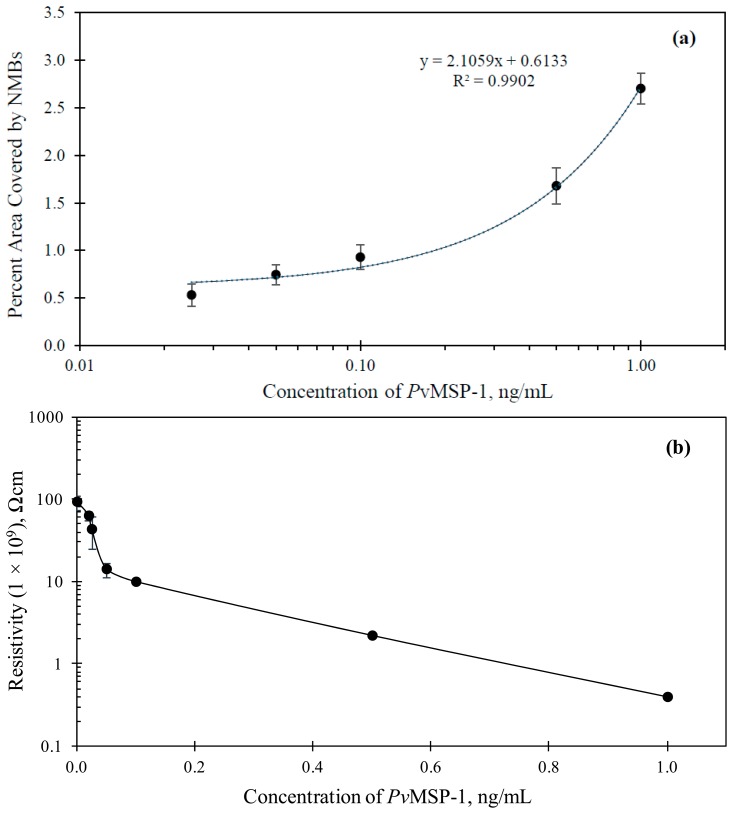
(**a**) Variation of percent area of the capture zone covered with NMBs against *Pf*MSP-1 concentrations and (**b**) Variation of the resistivity of the capture zone with the concentration of immobilized *Pf*MSP-1. The error bars in both graphs are also shown.

The assays with varying concentration of *Pf*MSP-1 in PBS showed a colored signal only at the second capture zone. Figure 5b shows the immunosensor for the detection of 10 ng/mL of *Pf*MSP-1 in PBS solution. Figure 6 shows the results of optical and electrochemical detection of *Pf*MSP-1 at the second capture zones, similarly to the study described in Figure 5 for *Pf*HRP-2 at the first capture zone. The correlation equation was *A*_pct_ = 0.6133 + 2.1059*x* (*R*^2^ = 0.9902). This has a good correlation and can be used to estimate the concentration of *Plasmodium vivax* in any sample tested. The visual detection limit of the assay thus obtained was 0.025 ng/mL. The lowest detection limit of *Pf*MSP-1 reported in the literature using immunoassay technique is 6.3 ng/mL [35]. The limit of detection using electrical measurements for the detection of *Pf*MSP-1 was 0.02 ng/mL (Figure 6b).

In another set of tests, a solution containing equal concentrations of *Pf*HRP-2 and *Pf*MSP-1 in PBS was prepared. The concentration of both antigens used for the study ranged from 0.025 to 10 ng/mL. In all the tests performed, NMBs were observed to be captured selectively on either capture zones. Figure 5c shows the immunosensor with specific NMBs at both capture zones.

This study showed that the NMB approach is selective in detecting mixed *Plasmodium falciparum* and *Plasmodium vivax* malaria parasites in solution. Antibodies specific to both *Pf*HRP-2 and *Pf*MSP-1 captured only the desired antigen at their designated capture zones. Signal quantification was obtained using both colorimetric and electrical methods both capture zones.

## 4. Conclusions

This work has demonstrated a highly sensitive method of detecting malaria parasites using NMBs as a label based on sandwich immunoassay protocol. Enhanced signal amplification was obtained by visual detection of 0.025 ng/mL of *Pf*HRP-2 in PBS within 1 min of testing. The method was also able to selectively capture *Pf*HRP-2 and *Pf*MSP-1 at different capture zones on the same immunosensor. Quantification of results were achieved by measuring the change in resistivity across the capture zone. This NMBs based detection eliminates the limitations imposed by MRDTs. The simplicity, speed, specificity, and ultra-sensitivity of this approach make it a potentially powerful tool for prompt diagnosis of malaria and detection of other infectious diseases.
